# Hantavirus transmission in the United States.

**DOI:** 10.3201/eid0303.970314

**Published:** 1997

**Authors:** R M Wells, J Young, R J Williams, L R Armstrong, K Busico, A S Khan, T G Ksiazek, P E Rollin, S R Zaki, S T Nichol, C J Peters

**Affiliations:** Centers for Disease Control and Prevention, Atlanta, Georgia, USA.

## Abstract

In 1996, investigation of a hantavirus pulmonary syndrome (HPS) outbreak in southern Argentina found evidence of person-to-person transmission of a hantavirus. The infection control ramifications of this finding led to this review of hantavirus epidemiology in the United States; the review suggests that Sin Nombre virus infection is rarely, if ever, transmitted from person to person and that existing guidelines for prevention of HPS remain appropriate for North America.


### Hantavirus Transmission in the United States

In 1996, investigation of a hantavirus pulmonary syndrome (HPS) outbreak in southern Argentina found evidence of person-to-person transmission of a hantavirus. The infection control ramifications of this finding led to this review of hantavirus epidemiology in the United States; the review suggests that Sin Nombre virus infection is rarely, if ever, transmitted from person to person and that existing guidelines for prevention of HPS remain appropriate for North America.

The public health significance of New World hantaviruses (genus Hantavirus, family Bunyaviridae) was first recognized in 1993 when healthy young adults in the southwestern United States contracted a mysterious pulmonary illness and died. The ensuing investigation identified a novel hantavirus, Sin Nombre virus (SNV), as the cause of a new syndrome, hantavirus pulmonary syndrome (HPS). A case-control study performed early in the outbreak showed that high density peridomestic rodent populations were a key risk factor for disease (1), a finding consistent with knowledge of Old World hantaviruses (including Puumala, Seoul, Hantaan, and Dobrava viruses), which can cause hemorrhagic fever with renal syndrome (HFRS) in humans who inhale aerosolized excreta from infected rodents (2). Thousands of episodes of HFRS occur in Eurasia each year, yet person-to-person transmission has never been reported. When person-to-person transmission of a hantavirus was suggested by the results of an HPS outbreak investigation in southern Argentina (3), we reviewed the epidemiology of HPS in the United States for evidence of nosocomial or secondary transmission.

Since 1993, a U.S. HPS registry has been maintained that includes surveillance case reports, environmental and epidemiologic case investigations, patients' medical records, and laboratory databases. This registry was used to identify the clusters of hantaviral infections shown in the Figure. A cluster was defined as the association of a patient with confirmed HPS with one or more contacts who had both laboratory evidence of SNV infection and a history of a febrile illness within 8 weeks of the onset of symptoms. Presence of serum immunoglobulin M (IgM) or IgG reacting with SNV antigen or immunohistochemical detection of SNV antigen in autopsy specimens was accepted as laboratory evidence of SNV infection. Eight weeks was selected as a conservative estimate of the incubation period on the basis of HFRS occurrence among United Nations (U.N.) military personnel within 7 days of arrival in (and as long as 46 days after departure from) a hantavirus-endemic area (4).

We identified five clusters (involving 12 patients) of hantaviral infections (Figure). The interval between cases was much shorter than 8 weeks in each instance, and contact between the linked patients from episodes 1, 3, and 4 occurred before patients were admitted to a hospital. All infected persons could have been exposed to rodent excreta in their home or work environments. Exposure risk was documented by results of rodent trapping in episodes 2, 3, and 4 (Table). In episodes 1 and 5, the men had been sleeping and working in rural environments where rodent infestation was noted, but trapping was not performed.

Viral products were amplified from the second of the two agricultural workers in episode 4 and from rodents captured at the ranch visited by the two men. The rodent and human viral sequences differed sufficiently to suggest that the second man's infection had been acquired elsewhere (S. St. Jeor, pers. comm.). Viral products could not be obtained from the other agricultural worker or from patients in the other clusters.

The U.S. case registry has on file 160 patients with clinical and laboratory evidence of HPS. Household or social contacts (n=320) of 40 of these HPS patients (including the HPS patients in the Figure) have been tested for antibodies that react with SNV antigen. Most contacts were household members or work colleagues of patients in whom HPS was diagnosed during the 1993 outbreak; testing sera from contacts of patients has not been routine in later years. Of these 320 household or social contacts, 310 had no serologic evidence of hantaviral infection,

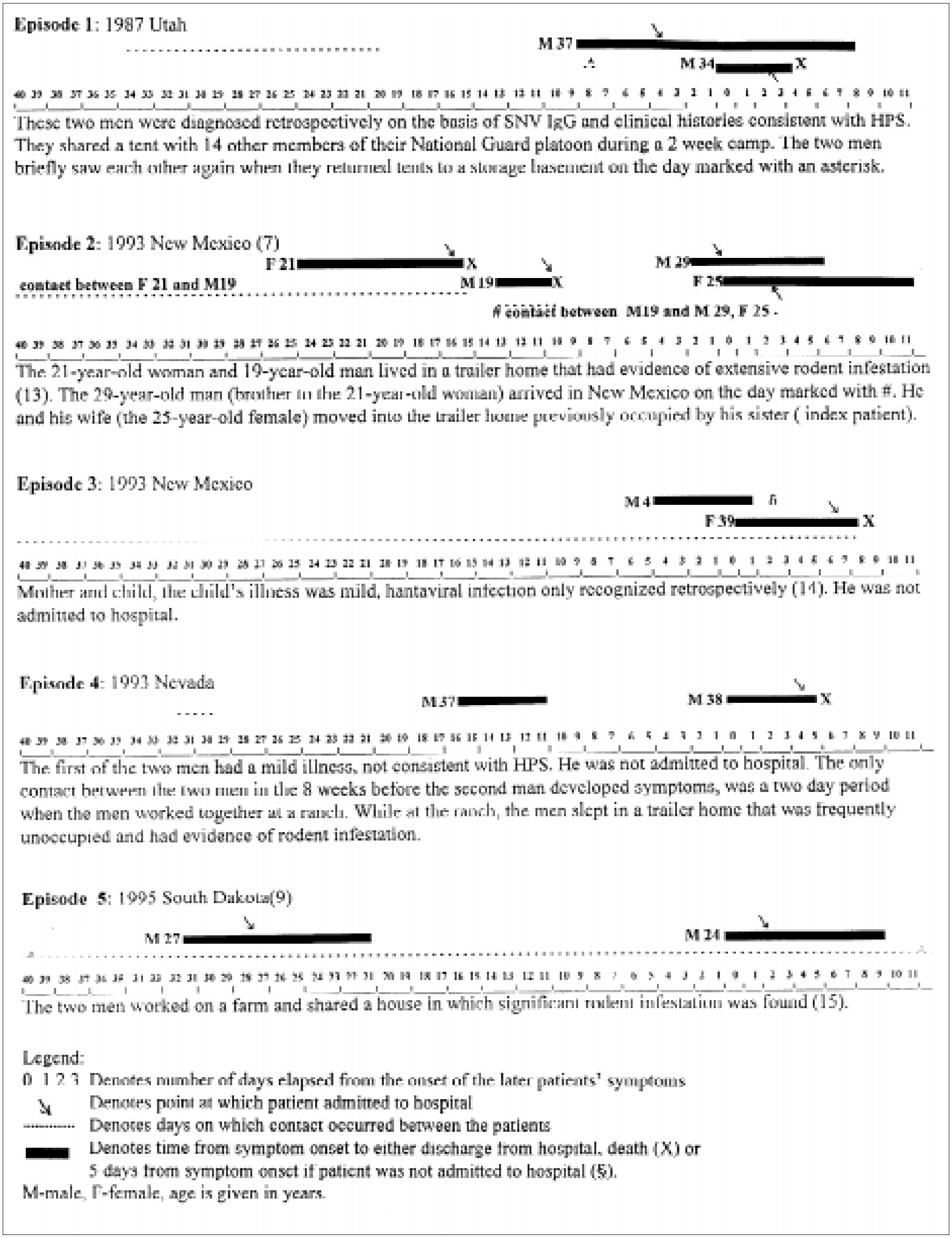

Figure. Clusters of hantaviral infections in the United States.*

*From a hantavirus pulmonary syndrome registry maintained by the Centers for Disease Control and Prevention three had IgG that reacted with SNV antigen (SNV-IgG) but no illness, one had SNV-IgG and was diagnosed with HPS retrospectively (episode 1, Figure), and six had IgM that reacted with SNV antigen (SNV-IgM). Four of the six contacts with SNV-IgM had confirmed cases of HPS (episodes 2 and 5), and two had a clinical course not consistent with that of HPS (episodes 3 and 4) (5). While person-to-person transmission cannot be ruled out, these data may simply reflect common exposure of the persons involved to infected rodents.

Nosocomial transmission of HPS has never been documented in the United States. In 1993, a survey of 396 health-care workers from New Mexico examined evidence of nosocomial transmission of HPS (6). Although 266 of these workers recalled recent exposure to a patient with HPS, none became ill with HPS or had serologic evidence of infection with SNV. Statistically, this estimate could have been obtained (with 95% confidence intervals) if true hantaviral seroprevalence among these workers was $\leq$ 3%. During the first month of the 1993 outbreak, health-care workers inconsistently adhered to precautions to avoid respiratory or blood contact. Small numbers of needlestick exposures and mouth-to-mouth resuscitation attempts did not result in documented hantavirus infection (F. Koster, pers. comm.). The only health-care worker in the United States to have a documented SNV infection had evidence of peridomestic rodent infestation and no known contact with other HPS patients. HFRS was not seen in attendants, nurses, doctors, or research workers involved in caring for more than 3,000 U.N. military personnel who contracted HFRS in Korea during the early 1950s (2).

In contrast, one hospital receptionist and five doctors were among the 20 patients involved in the HPS outbreak in southern Argentina (3). Only two of the six recalled seeing rodents in the 6 weeks before onset of HPS; and when trapping was performed in the homes of four of these persons, no evidence of rodent infestation was found and no rodents were captured (J. Mills, pers. comm.).

While HPS in Argentinean health-care workers was unexpected, clustering of HPS patients is in keeping with rodent-borne zoonoses. Small localized clusters of HFRS were not uncommon among U.N. troops in Korea, with 1- to 3-week intervals between the onset of symptoms in the first and last patients (7). A cross-sectional study of hantavirus antibody prevalence in rural China identified localized clustering of HFRS patients, but the association between patients with HFRS was not statistically significant (8). Outbreaks of HFRS among laboratory workers also is well recognized. Epidemiologic investigation of one such outbreak found no hantaviral infections in household contacts of 113 persons who became infected after entering a building that housed a laboratory colony of infected rodents (9).

Electron microscopy and immunohistochemical studies of tissues from HPS patients suggest why these viruses may have such a low propensity to cause secondary hantaviral infections in humans. Despite immunohistochemical examination of tissues from HPS patients showing high levels of hantaviral antigens in the pulmonary microvasculature, mature virus particles are rarely visualized (10). Disease in humans appears to result from an overwhelming immunologic response after exposure to hantaviral antigens. The direct cytopathic effects of virus on host cells (as seen in some other devastating viral infections, such as that caused by Ebola virus) is not a feature of HPS. Differences in infectiousness could reflect the extent of viral replication and spread before the immune response becomes active or failure of the immune response to promptly eradicate virus.

Although infected rodents develop antibodies that react with hantaviral antigens, they do not have the immunologically mediated manifestations characteristic of HPS in humans. Rodents can continue to excrete virus in their

Table. Seropositivity of humans and rodents from episodes in which clustering of human hantaviral infections were identified in the United States

| Episode | Household contacts No. Positive (No. tested) | Nonhousehold contacts No. Positive (No. tested) | Site contacts[a] No. Positive (No. tested) | Rodents No. trapped[b] | Seropositive rodents Positive / No. tested (%) |
|---|---|---|---|---|---|
| 1 | 0 (5) | 0 (19) | 0 (0) | nd | nd |
| 2 | 3 (9) | 0 (11) | 0 (0) | 40 | 8/27 (30) |
| 3 | 1 (7) | 0 (0) | 0 (0) | 87 | 15/41 (37) |
| 4 | 1 (2) | 0 (20) | 0 (9) | 80 | 7/80 (9) |
| 5 | 1 (1) | 0 (2) | 0 (0) | nd | nd |

[a]Persons with exposure to the home or worksite of the index patient but without exposure to the patient
[b]Trapping occurred in and around index patient's home/worksite
nd= not done urine, saliva, and feces for prolonged periods despite the presence of serum antibodies. Hantaviruses have been isolated (albeit with difficulty) from infected rodents but not from tissues of HPS patients. Viral RNA is plentiful, however, and frequently can be amplified by reverse transcriptase and polymerase chain reaction (RT-PCR) from the blood and tissues of HPS patients. When the amino acid composition of viral proteins from HPS patients (coded for by these RT-PCR products) from southern Argentina was compared with that of other New World hantaviruses, differences varied from 13.6% to 23.9% for fragments of the G2 protein regions and from 8.5% to 12.5% for a 528 base pair fragment of the amino terminal region of the nucleocapsid protein (11). Thus, SNV (the etiologic agent responsible for most hantaviral infections in the United States) is phylogentically related to, but clearly distinct from, the Argentinean hantavirus, provisionally named Andes virus. The rodent host for the southern Argentinean virus is *Oligoryzomys longicaudatus*, a species not found in North America (12).

Milder disease was not a feature of the southern Argentinean outbreak; but interestingly, two patients with mild disease were identified in our search for HPS clusters. A small amount of mild disease resulting from SNV infection probably goes unrecognized in the United States. For example, in episodes 3 and 4, a diagnosis of febrile illness in the first patients was made only after the more severe course of HPS in the later patients led to testing for hantaviral antibodies. Hantaviral antibodies in sera from only 78 U.S. residents (in addition to the 160 confirmed patients) were identified by the Centers for Disease Control and Prevention, despite testing sera from approximately 7,000 U.S. residents as part of diagnostic work-ups, surveys of potential high-risk populations, and blinded population surveys.

Whether the secondary transmission of HPS observed in Argentina is a feature peculiar to hantaviruses in southern Argentina or a rare interaction between host and virus pertinent to all members of the genus is not known. In outbreaks associated with some highly pathogenic viruses (e.g., Ebola virus), “super-spreaders” (patients who transmit virus to many persons) have been an important factor in ongoing transmission of disease (A.S. Khan, pers. comm.). It is unlikely that such a phenomenon was solely responsible for the 1996 outbreak of HPS in southern Argentina, however, because multiple chains of transmission appear to have occurred (3).

This investigation highlights the importance of maintaining surveillance systems for emerging diseases such as HPS. Five clusters of patients in the U.S. HPS case registry were not unexpected on the basis of previous experience with hantaviral diseases. Although person-to-person transmission of SNV cannot be completely excluded, the clusters were consistent with the exposure of multiple persons to infected rodents. Thorough future investigation of clusters of SNV-infected patients will be important, but now it appears that existing recommendations have been sufficient to prevent secondary transmission of HPS in the United States.

### Acknowledgments

We thank John O'Connor for his editorial comments and the following people for their assistance in clarifying the many questions raised during investigation of the five clusters: B. Tempest, F. Koster, G. Simpson, W. Frampton, J. Childs, S. St Jeor, and J. Tappero.

**Rachel M. Wells, Joni Young, R. Joel Williams, Lori R. Armstrong, Kristi Busico, Ali S. Khan, Thomas G. Ksiazek, Pierre E. Rollin, Sherif R. Zaki, Stuart T. Nichol, and C.J. Peters**

Centers for Disease Control and Prevention, Atlanta, Georgia, USA

### References

1. Zeitz PS, Butler JC, Cheek JE, Samuel MC, Childs JE, ShandsLA, et al. A case-control study of hantavirus pulmonary syndrome during an outbreak in the southwestern United States. J Infect Dis 1995;171:864-70.
2. Lee HW, van der Groen G. Hemorrhagic fever with renal syndrome. Prog Med Virol 1989;36:62-102.
3. Wells RM, Sosa Estani S, Yadon ZE, Enria D, Padula P, Pini N, et al. An unusual hantavirus outbreak in southern Argentina: person-to-person transmission? Emerg Infect Dis 1997;3:171-4.
4. Powell GM. Hemorrhagic fever: a study of 300 cases. Medicine (Baltimore) 1954;33:97-153.
5. Centers for Disease Control and Prevention. Case defnitions for infectious conditions under public health surveillance. MMWR Morb Mort Wkly Rep 1997;46:RR-10.
6. Vitek CR, Breiman RF, Ksiazek TG, Rollin PE, McLaughin JC, Umland ET, et al. Evidence against person-to-person transmission of hantavirus to health care workers. Clin Infect Dis 1996;22:824-6.
7. Gauld RL, Craig JP. Epidemiological pattern of localized outbreaks of epidemic hemorrhagic fever. American Journal of Hygiene 1954;59:32-8.

8. Ruo SL, Li YL, Tong Z, Ma QR, Liu ZL, Tang YW, et al. Retrospective and prospective studies of hemorrhagic fever with renal syndrome in rural China. J Infect Dis 1994;170:527-34.
9. Kulagin CM, Fedorova H, Ketiladze EC. Laboratory outbreak of hemorrhagic fever with a renal syndrome (clinico-epidemiological characteristics). Journal of Microbiology, Epidemiology and Immunology 1962;33:121-6.
10. Zaki SR, Greer PW, Coffield LM, Goldsmith CS, Nolte KB, Foucar K, et al. Hantavirus pulmonary syndrome. Pathogenesis of an emerging infectious disease. Am J Pathol 1995;146:552-79.
11. Lopez N, Padula P, Rossi C, Lazaro ME, Franze-Fernandez MT. Genetic identification of a new hantavirus causing severe pulmonary syndrome in Argentina. Virology 1996;220:223-6.
12. Musser GG, Carleton MD. Family Muridae. In: Wilson DE, Reeder DM, editors. Mammal species of the world, a taxonomic and geographic reference. 2nd ed. Washington (DC): Smithsonian Institution 1993:501-755.
13. Duchin JS, Koster FT, Peters CJ, Simpson GL, Tempest B, Zaki S, et al. Hantavirus pulmonary syndrome: a clinical description of 17 patients with a newly recognized disease. N Engl J Med 1994;330:949-55.
14. Armstrong LR, Bryan RT, Sarisky J, Khan AS, Rowe T, Ettestad PJ, et al. Mild hantaviral disease caused by Sin Nombre virus in a four-year-old child. Pediatr Infect Dis J 1995;14:1108-10.
15. Centers for Disease Control and Prevention. Hantavirus pulmonary syndrome—United States, 1995 and 1996. MMWR Morb Mort Wkly Rep 1996;45:291-5.
